# Evaluation of a *MdMYB10*/*GFP43* fusion gene for its suitability to act as reporter gene in promoter studies in *Fragaria vesca* L. ‘Rügen’

**DOI:** 10.1007/s11240-017-1229-0

**Published:** 2017-05-16

**Authors:** Yehia A. Khidr, Henryk Flachowsky, Christian Haselmair-Gosch, Jana Thill, Silvija Miosic, Magda-Viola Hanke, Karl Stich, Heidi Halbwirth

**Affiliations:** 1 Julius Kühn-Institut, Federal Research Centre for Cultivated Plants, Institute for Breeding Research on Fruit Crops, Pillnitzer Platz 3a, 01326 Dresden, Germany; 2 Plant Biotechnology Department, Genetic Engineering and Biotechnology Research Institute, University of Sadat City, P.O. Box 32897, 5th Zone, Sadat, Egypt; 3 Technische Universität Wien, Institute of Chemical, Environmental and Biological Engineering, Getreidemarkt 9, 1060 Vienna, Austria

**Keywords:** *Fragaria vesca*, Anthocyanin, MYB10 transcription factor, *Flavonoid 3′-hydroxylase* (*F3′H*), *Chalcone 3-hydroxylase* (*CH3H*), Reporter gene, CaMV *35S* promoter, Green fluorescent protein (GFP)

## Abstract

A *Malus domestica MdMYB10* transcription factor gene was previously used as visible marker for successful plant transformation. We combined the *MdMYB10* transcription factor gene with a *GFP* gene to test its viability as a non-destructive, visual, double reporter system for functional promoter studies in transgenic strawberry plants. The *GFP* gene was fused to *MdMYB10* to provide evidence for promoter activity in red colored cells of transformed plant tissue and to exclude artefacts resulting from stress response or due to other environmental cues. To test this system in a first approach, we evaluated the *MdMYB10-GFP43* construct in transgenic strawberries in combination with two constitutive promoters of varying strength, the strong CaMV *35S* promoter and a weak *flavonoid 3′-hydroxylase* (*F3′H*) promoter isolated from the ornamental plant *Cosmos sulphureus. Agrobacterium tumefaciens* mediated transformation of *Fragaria vesca* with the *MdMYB10-GFP43* construct combined with the CaMV* 35S* or *F3′H* promoter sequences resulted in the regeneration of 6 and 4 transgenic lines, respectively. A complete red coloration of all plant organs was found in four out of six transgenic lines harboring the *35S-MdMYB10-GFP43* construct. Less red coloration of plant organs was found for lines transformed with the *F3′H-MdMYB10-GFP43* construct. The *MdMYB10* gene shows only limited suitability as a reporter gene for promoter studies in strawberries because weak promoter activity is difficult to distinguish, particularly in tissues showing a strongly colored background such as green leaves. GFP specific fluorescence signals were detectable neither in tissue strongly expressing *MdMYB10* nor in green tissue of any transgenic line. The reason for this remained unclear but it can be excluded that it was due to incorrect splicing.

**Electronic supplementary material:**

The online version of this article (doi:10.1007/s11240-017-1229-0) contains supplementary material, which is available to authorized users.

## Introduction

Genetic modification of plants offers a palette of tools which nowadays are of particular importance for modern plant science and breeding. They are very helpful in functional genomics, the improvement of quality and quantity of agronomical traits and the production of desirable components for mass production (Moose and Mumm 2008). However, in the forefront of breeding, target genes, suitable promoters or other regulatory sequences need to be discovered and functionally characterized. This is usually done in model plants as this has numerous advantages for forward and reverse genetics. These include their small genome size, their short generation time, their small plant size allowing experiments with hundreds of plants under lab conditions and their close genetic relationship to commercially important crops. *Arabidopsis thaliana* (L.) Heynh. is the most widely-studied plant so far and serves as a model system for identifying genes, determining their functions and understanding the complex processes involved in plant growth and development (Initiative 2000; Rhee et al. 2003). The woodland strawberry (*F. vesca* L.) is gaining increased attention as a model system for the Rosaceae plant family (Slovin et al. 2009; Zhang et al. 2014) which includes a number of economically important fruit species such as apple, pear, quince, peach, apricot and the cultivated octoploid garden strawberry *Fragaria* × *ananassa* Duch. The woodland strawberry seems to be ideal for functional genomics studies because of its diploid genome with 2n = 2x = 14 chromosomes and a genome size of 240 Mbp (Shulaev et al. 2011), the short seed to seed cycle of 3.0–3.5 months, its ease of propagation from seed and clones in small 7.5 cm plastic pots, the high efficiency of *Agrobacterium*-mediated transformation, the rapid in vitro regeneration and the availability of the complete genome sequence and other modern genetic tools (Guidarelli and Baraldi 2015; Lin-Wang et al. 2014). However, some tools for studying gene and promoter function are still needed or need improvement (Carvalho and Folta 2017; Gunadi et al. 2016). For example, good reporter genes for promoter analyses that allow a non-destructive easily visible evaluation of tissues without the need for fluorescence or light imaging would be very helpful. Genes triggering tissue coloration that is easily detectable by visual means, like the *MdMYB10* gene of apple, a MYB-type transcription factor leading to increased production of anthocyanin (Allan et al. 2008; Ban et al. 2007; Espley et al. 2007; Takos et al. 2006), were several times suggested as good candidates (Hoffmann et al. 2006; Rosellini 2012).

Anthocyanins are a class of flavonoid pigments providing red, blue or purple pigmentation to fruits, flowers, foliage, roots and stems and are suggested to contribute to human health (van Nocker et al. 2012; Würdig et al. 2014). Recently it was shown that anthocyanin formation can be used as a potential visual selection marker during plant transformation (Kortstee et al. 2011) as an alternative to removable marker gene systems or chemically selectable markers, such as kanamycin resistance. Kortstee et al. (2011) transformed the *MdMYB10* together with a mutant *MdMYB10* promoter allele from the apple cv. ‘Red Field’ into apple, strawberry and potato as model crop species. Red colored calli, red shoots and red well-growing apple and strawberry plants reliably indicated successful transformation events, although a small number of falsely negative regenerates were described as well (Kortstee et al. 2011).

With this in mind, we constructed a new *MdMYB10*/*GFP43* fusion gene intended to be useful for the non-destructive discovery of protein expression by simple visual control on red coloration, which can be simultaneously double-checked by green fluorescence. The *MdMYB10*/*GFP43* fusion gene was placed under the regulation of the strong constitutive CaMV 35S promoter and a second, constitutive plant-derived promoter [*flavonoid 3′-hydroxylase* (*F3′H*) *promoter*] respectively. The resulting transgenic plants were intensively evaluated to test the suitability of the novel reporter gene for promoter studies in the model plant *Fragaria vesca*.

## Materials and methods

### Plant material

For the isolation of the 5′-flanking regulatory regions closed buds with 5 mm length of *Cosmos sulphureus* cv. ‘Sunny Goldgelb’ (Austrosaat, Vienna, Austria) were collected during summer 2012, frozen in liquid nitrogen and stored at −80 °C until use. For plant transformation and promoter evaluation the S10 inbred line of the diploid woodland strawberry (*Fragaria vesca* L.) cv. ‘Rügen’ was used.

### Isolation and analysis of the 5′-flanking regions

Genomic DNA was isolated from closed buds according to Aldrich and Cullis (1993). The 5′-flanking regions were isolated using the GenomeWalker™ Universal Kit (Clontech, Saint-Germain-en-Laye, France) according to the manufacturer’s instructions. The first primer pair was designed based on the *F3′H* sequence (GenBank: FJ216426) (Schlangen et al. 2010). DNA-fragments were isolated and ligated into the vector pCR^®^2.1-TOPO (Invitrogen, Paisley, UK) and transformed in *E. coli* TOP10 (Invitrogen, Paisley, UK). Plasmids were isolated using the Wizard Miniprep Kit (Promega, Mannheim, Germany) and sequenced by StarSEQ (Mainz, Germany). Further primers for genome walking were designed from the sequences obtained (Suppl. Table S1). Finally, a putative *F3′H promoter* sequence containing 1712 bp of the 5′-flanking region (GenBank: KU508433) was obtained with the primer pair Pro.F3′H. A putative *chalcone 3-hydroxylase* (*CH3H) promoter* sequence was obtained in a similar way (GenBank: KU508432) (Supplemental material).

The transcription start site (TSS) in the 5*′*-flanking region of the *F3′H* was predicted by using the TSSP software (Softberry, http://linux1.softberry.com), and the cis-acting regulatory elements were analysed by using the PlantCARE software (http://bioinformatics.psb.ugent.be/webtools/plantcare/html/).

### Preparation of the binary plasmid vector constructs

The binary plasmid vectors p9N::*35S-MdMYB10-GFP43* and p9N::*F3′H-MdMYB10-GFP43* were provided by DNA Cloning Service e.K. (Hamburg, Germany). The *MdMYB10* coding region of the red leaved apple hybrid TNR 31-35 of *Malus sieversii* var. *sieversii* f. *niedzwetzkyana* (Espley et al. 2007, 2009; Würdig et al. 2014), including its first intron and a codon usage optimized version for the expression in dicotyledons of the red-shifted smRS-GFP gene (Davis and Vierstra 1998) containing the intron of the potato *ST-LS1* gene, were used for the construction of the *MdMYB10*/*GFP43* fusion gene. A p9N::*35S-MdMYB10-GFP43* vector was constructed using the p9N-*35S* binary plasmid vector (DNA Cloning Service) that contains the *nptII* selectable marker gene driven by the *nopaline synthase* promoter from *A. thaliana*. The p9N::*F3′H-MdMYB10-GFP43* vector was obtained by exchanging the CaMV 35S promoter of p9N-*35S* for the *F3′H* promoter (Gene bank: KU508433).

### Plant transformation

Plant transformation was performed as described recently (Fischer et al. 2014) using the *A. tumefaciens* strain EHA105 (Hood et al. 1993), with each plant harboring one of the binary plasmid vectors p9N::35S-MdMYB10-GFP43 and p9N::F3′H-MdMYB10-GFP43. Wounded leaf discs of *F. vesca* cv. ‘Rügen’ were inoculated with the A. tumefaciens strain EHA105 carrying one of the two binary plasmid vectors differing in the promoter controlling the fusion gene (p9N::*35S-MdMYB10-GFP43*, and p9N::*F3′H-MdMYB10-GFP43*). Selection of putative transgenic plants was performed on regeneration medium containing 500 mg l^−1^ timetin and 300 mg l^−1^ kanamycin. The explants were transferred into light after 3 weeks and subcultured every 3 weeks. Regenerated plantlets were transferred onto hormone free MS regeneration medium and transferred to soil as soon as plantlets had produced sufficient roots. They were acclimated in mini greenhouses where they were grown for 3 weeks in 5 cm plastic pots supplemented with potting compost and perlite. Subsequently they were grown under normal greenhouse conditions in 8 and 12 cm plastic pots.

Using the vector p9N::*35S-MdMYB10-GFP43*, one transformation experiment with 580 leaf explants resulted in 14 regenerated plants, six of which survived the entire selection process. These six plants were further handled as putative transgenic plants and labeled with F-133, F-134, and F-143 to F-146. Using the vector p9N::*F3′H-MdMYB10-GFP43*, a single transformation experiment with 260 leaf explants resulted in seven regenerated plants. Four out of these plants survived and were regarded as putative transgenic plants (F-154, and F156 to F-158).

### PCR and Southern blot analysis

Analysis of integrated T-DNA in the plant genome was performed by PCR. Genomic DNA was extracted from 50 to 60 mg leaf tissues using the DNeasy Plant Mini Kit (Qiagen, Hilden, Germany). Transferred DNA sequences including *nptII, MdMYB10, GFP43, MdMYB10::GFP43*, and the housekeeping gene *elongation factor 1-alpha* (*EF1α*) were amplified by PCR using appropriate primer pairs listed in Table 1. The PCR reaction was performed in 25 µl containing 5–50 ng of template DNA, 1× DreamTaq™ buffer, 0.2 mM dNTPs, 0.5 µM of each primer and 0.5 unit DreamTaq™ DNA polymerase (MBI Ferments, St. Leon-Roth, Germany). The PCR program was performed as follows: Initial denaturation at 94 °C for 2 min, followed by 30 cycles of denaturation (94 °C for 30 s), annealing (53–63 °C for 1 min) and extension (72 °C for 1 min) and a final extension at 72 °C for 7 min.

**Table 1:** Primers used for molecular evaluation of the transgenic strawberry plants, position in the construct is shown in Fig. 1

Gene	Primer: sequence (5*′*–3*′*)	Annealing (°C)	Amplicon size (bp)	
			Genomic	cDNA
nptII	nptII_F: ACAAGATGGATTGCACGCAGG nptII_R AACTCGTCAAGAAGGCGATAG	58	780	780
MdMYB10a	MYB_F^a^:** C**A**AA**GCAGGCTTAAACAGGTG MYB_R: TAAGACCTCAGCCCCAAAAAT	60	296	296
GFP43	GFP_F: CTTTCAAGGACGACGGAAATTA GFP_R: GATTGTCAGGGAGAAGAACTGG	60	300	300
EF1*α*	EF1-F: ATTGTGGTCATTGGYCAYGT EF1-R: CCAATCTTGTAVACATCCTG	58	800	700
MdMYB10::GFP43	MYB_ATG: ATGGAGGGATATAACGAAAACCTG GFP_RR: CATCCATTCCATGAGTGATACC	63	1974	1462

^a^Primer was available and originally designed for *MdMYB10a*, which is a homolog of *MdMYB10*. This primer is not optimal, because of three mismatches at the 5′-end (written in bold) and eight bases at the 5′-end which are located in the intron sequence (underlined). However, the primer was initially tested in combination with MdMYB10_R and successful on DNA and cDNA

**Fig. 1:**
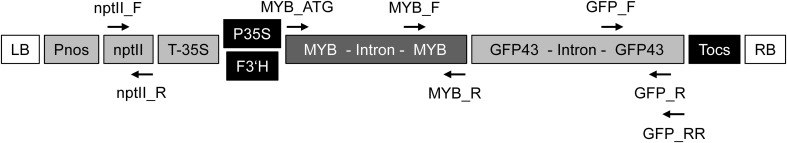
Schematic representation of T-DNA construction of the binary plasmids p9N::35S-MdMYB10-GFP43 and p9N::F3′H-MdMYB10-GFP43. LB and RB, T-DNA left and right border sequences; Pnos, promoter sequence of the nopaline synthase gene; nptII, CDS of the *nptII* selectable marker gene; T-35S, terminator sequence of the *35S* Cauliflower mosaic virus gene; P35S, promoter sequence of the *35S* Cauliflower mosaic virus gene; F3*′*H, promoter sequence of the *F3′H* gene; MYB-Intron-MYB, synthetic CDS of the *MdMYB10* gene still containing its first native intron; GFP43-Intron-GFP43, synthetic CDS of the *GFP43* gene interrupted by the *STLS1* intron; Tocs, terminator sequence of the octopine synthase gene. Positions of primers are marked by *arrows*. Primer names are given as abbreviations. The full names are given in Table 1

Analysis of integrated T-DNA copies in the plant genome was performed by Southern hybridization as recently described (Fischer et al. 2014). The genomic DNA was extracted from 100 mg plant leaf tissue using a modified cetyl trimethyl ammonium bromide (CTAB) extraction protocol. 10 µg DNA were incubated with 100 U of *Bam*HI (MBI Ferments, St. Leon-Roth, Germany) at 37 °C overnight. The cleaved DNA was separated on a 1% agarose gel and transferred onto a nylon membrane (Roche Diagnostics, Mannheim, Germany). Digoxygenin-labeled probes for hybridization were amplified using the forward and reverse primers on the coding region of the *nptII* gene. Hybridization and detection were performed using the ECF-Random-Prime-Labeling and Detection Kit (Amersham Biosciences, Freiburg, Germany) according to the manufacturer’s manual.

### Gene expression analysis

Analysis of transcript abundance was performed by (RT)-PCR. Total RNA was extracted from 50 mg plant tissues using InviTrap^®^ Spin Plant RNA Mini Kit 1012 (STRATEC Biomedical AG, Birkenfeld, Germany). 1 µg of total RNA was used for the reverse transcription using the RevertAid™ First Strand cDNA Synthesis Kit (Thermo Scientific, Braunschweig, Germany). The generated cDNA was used as a template for PCR, which was performed using the same primers and conditions as described above. Full length amplification of the *MdMYB10*/*GFP43* fusion gene was performed using the Phusion DNA Polymerase (ThermoFisher Scientific, Schwerte, Germany) in a final volume of 20 µl using primers MYB_ATG and GFP_RR described in Table 1 and Fig. 1. The PCR reaction was performed as follows: Initial denaturation at 98 °C for 30 s, followed by 30 cycles of denaturation (98 °C for 10 s), annealing (63 °C for 30 s) and extension (72 °C for 1 min) and a final extension at 72 °C for 7 min.

### Phenotypic evaluation of transgenic strawberries

Leaves, crowns, flowers (sepal, petal, stamen, carpel), and fruits of all transgenic lines in comparison to the wild type (wt) line were evaluated and phenotypic characteristics were documented by photos. The examination for GFP fluorescence was done using a Zeiss Axioskop 135 microscope (TSO Thalheim Spezialoptik GmbH, Germany). For this, transgenic plant leaves, stems, flowers, and flower buds and the non-transgenic control tissues were cut into small pieces and mounted in water on glass microscope slides for inspection.

## Results

### Isolation of the 5′-flanking region of the *F3′H* from *C. sulphureus*

Using the sequences of the *F3′H* cDNA clone (GenBank: FJ216426) specific primers were designed and used as a starting point for the stepwise *de novo* sequencing of the 5′-flanking region of *F3′H* from genomic DNA obtained from buds of *C. sulphureus*. A putative *F3′H* promoter region of 1.7 kb length was obtained by genome walking. An NCBI blast of the *F3′H* promoter region did not show any similarities with known sequences. Comparison of the putative *F3′H* promoter regions of *C. sulphureus* (GenBank: KU508433) and a *Fragaria vesca F3′H* (GenBank: KC708488) showed only around 40% correlation and stretches of identic sequence were below eight nucleotides. The isolated 5*′*-flanking region was of comparable size to other *F3′H* promoter regions available in the NCBI data base for *Antirrhinum majus* (1529 bp; JF309098), *Malus* hybrid cultivar (1393 bp; KT288226), *Vitis vinifera* (952 bp, KT216255) and *Tulipa fosteriana* (1900 bp; KF146886, 1901 bp; KF751606).

The *in silico* analysis of the 5*′*-flanking region of the *F3′H* revealed a putative TSS 44 bp upstream of the translation initiation codon ATG. Beside the typical TATA-box (63 bp upstream of ATG) and CAAT-box (75 bp upstream of ATG), several other cis-acting regulatory elements were predicted with the PlantCARE software tool. These included light responsive elements (e.g. Box I, G-Box, I-box, MNF1, Sp1, and the motifs ACE, CATT, TCT, GATA) besides a number of other putative regulatory elements, e.g. ABRE (abscisic acid responsiveness), Box-W1 (fungal elicitor responsive element), CGTCA-motif (involved in the methyl jasmonate responsiveness), EIRE (elicitor responsive element), ERE (ethylene responsive element), HSE (heat stress responsiveness), LTR (low temperature responsiveness), MBS (MYB binding site involved in drought inducibility), TC-rich repeats (defense and stress responsiveness), WUN-motif (wound responsive element) or Skn-1 motif (required for endosperm expression).

### Molecular evaluation of putative transgenic strawberry plants

All putative transgenic plants F-133, F-134, and F-143 to F-146 transformed with p9N::35S-MdMYB10-GFP43 and the plants F-154, F-156, F-157, and F-158 with p9N::F3′H-MdMYB10-GFP43 were evaluated for the presence of transgenic DNA sequences by PCR with genomic DNA as template. The quality of the isolated DNA was tested using the primers EF1-F and EF1-R which are specific for the housekeeping gene *EF1α*. A fragment with a size of 800 bp could be amplified for each sample (Table 2). Subsequently all samples were PCR tested on the presence of the transferred gene sequences using the primers nptII_F/R for *nptII*, GFP_F/R for *GFP43*, and MdMYB10a_F/R for *MdMYB10*, which resulted in amplification products of 780, 300 and 296 bp, respectively (all primers are shown in Table 1). Genomic DNA from all ten plants was positively tested for the presence of all three transgenes (Table 2). Subsequently, these ten plants were propagated vegetatively to produce transgenic clones.

**Table 2:** PCR based evaluation of leaf tissue of the transgenic strawberry lines

Plasmid vector	Genotype	Genomic DNA				cDNA			
		EF1α _(800 bp)_	nptII _(780 bp)_	MdMYB10_(296 bp)_	GFP43_(300 bp)_	EF1α _(700 bp)_	nptII _(780 bp)_	MdMYB10 _(296 bp)_	GFP43_(300 bp)_
	‘Rügen’	+	n. d	n. d	n. d	+	n. d	n. d	n. d
p9N::35S-MdMYB10-GFP43	F-133	+	+	+	+	+	+	+	+
	F-134	+	+	+	+	+	+	+	+
	F-143	+	+	+	+	+	+	+	+
	F-144	+	+	+	+	+	+	+	+
	F-145	+	+	+	+	+	+	+	+
	F-146	+	+	+	+	+	+	+	+
p9N::F3*′*H-MdMYB10-G FP43	F-154	+	+	+	+	+	+	+	+
	F-156	+	+	+	+	+	+	n. d	n. d
	F-157	+	+	+	+	+	+	+	+
	F-158	+	+	+	+	+	+	+	+

n. d. not detected

The integration of the T-DNA into the strawberry genome of the ten transgenic lines was evaluated by Southern hybridization. *NptII* specific hybridization signals were detected in all samples (data not presented).

### Transcript analysis

The presence of transcripts in leaf tissue was tested by RT-PCR using the same primers for *nptII, MdMYB10, GFP43* and *EF1α* as described above. For all lines (transgenic and wild type) a 700 bp fragment of *EF1α* was amplified as expected for uncontaminated cDNA. In none of samples the 800 bp fragment for genomic DNA was detected. Transcripts of *nptII, MdMYB10* and *GFP43* could only be detected in leaf samples of nine out of the ten transgenic lines (Table 2). No RT-PCR products were detectable for *MdMYB10* and *GFP43* for the sample of F-156.

**Fig. 2:**
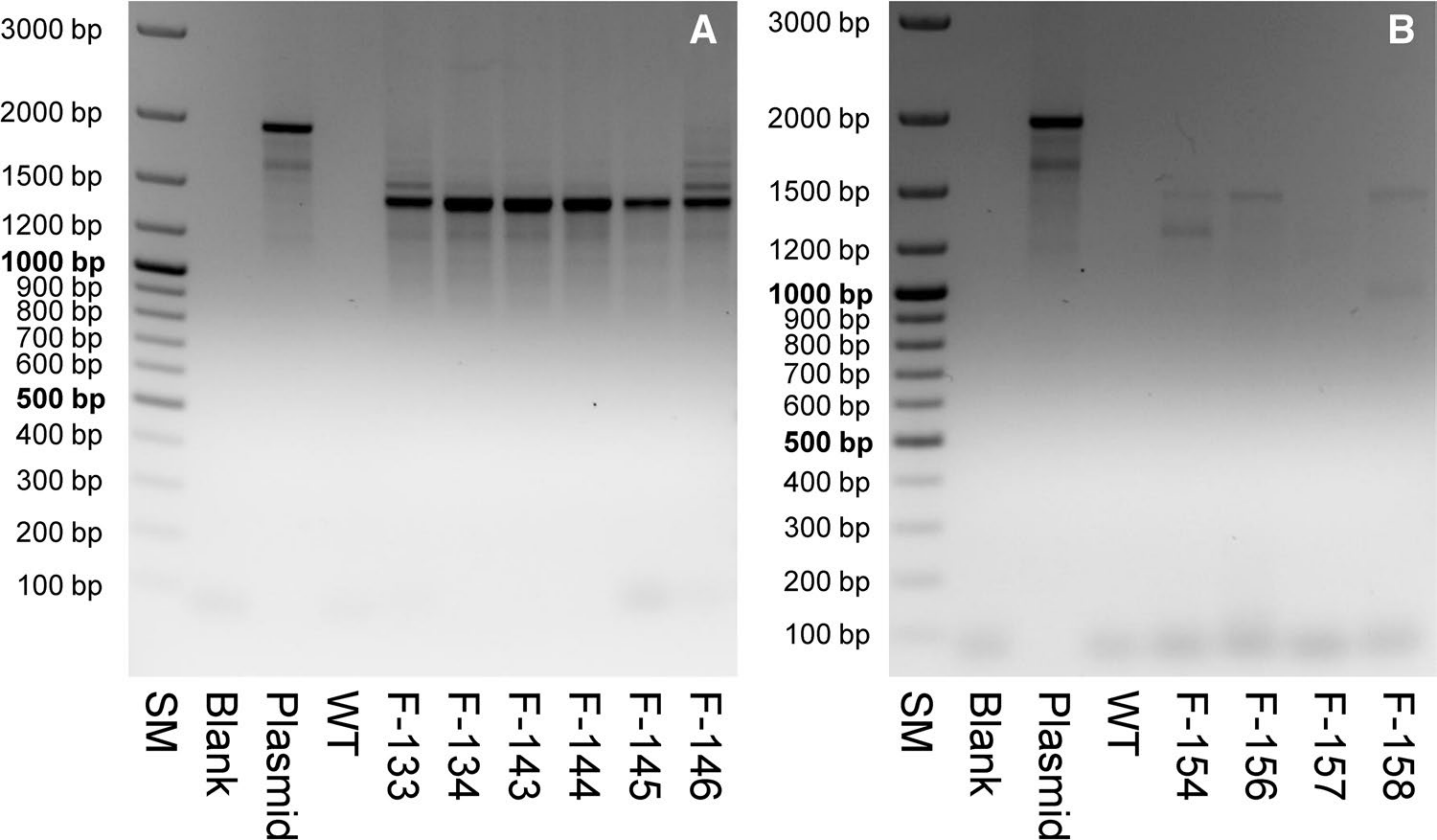
Evaluation of transcription of the *MdMYB10*/*GFP43* fusion gene using the primers MdMYB10_ATG and GFP43_RR. **a** For genomic DNA and plasmid DNA of the transformation vector a fragment of 1974 bp is expected. **b** For cDNA of the transgenic strawberry clones a fragment of 1462 bp is expected. SM, Gene Ruler™ 100 bp DNA Ladder Plus (ThermoFisher Scientific, Schwerte, Germany); Blank, H_2_O instead of DNA used as negative control; Plasmid, DNA of the plasmid used for plant transformation as positive control; WT, cDNA of the non-transgenic wild type cv. ‘Rügen’ used as negative control; F-133 to F-146, cDNA of the transgenic strawberry lines transformed with p9N::35S-MdMYB10-GFP43; F-154 to F-158, cDNA of the transgenic strawberry lines transformed with p9N::F3′H-MdMYB10-GFP43

Full length transcription of the *MdMYB10*/*GFP43* fusion gene was initially tested on leaf tissue using the primers MYB_ATG and GFP43_RR. The primer pair flanks the *MdMYB10*/*GFP43* fusion gene (Fig. 1) and amplifies a 1974 bp fragment on genomic DNA. After successful splicing of the two introns, the same primer pair amplifies a fragment of 1462 bp on cDNA. A PCR fragment of strong intensity and a size of <1.5 kbp was detected in all samples of plants transformed with p9N::35S-MdMYB10-GFP43 (Fig. 2a). A very faint band of identical size could be detected for cDNA samples of three out of the four lines (F-154, F-156, and F-158) transformed with p9N::F3*′*H-MdMYB10-GFP43. No fragment was detectable for the sample of F-157 (Fig. 2b).

### Phenotypic evaluation of transgenic strawberry lines

**Fig. 3:**
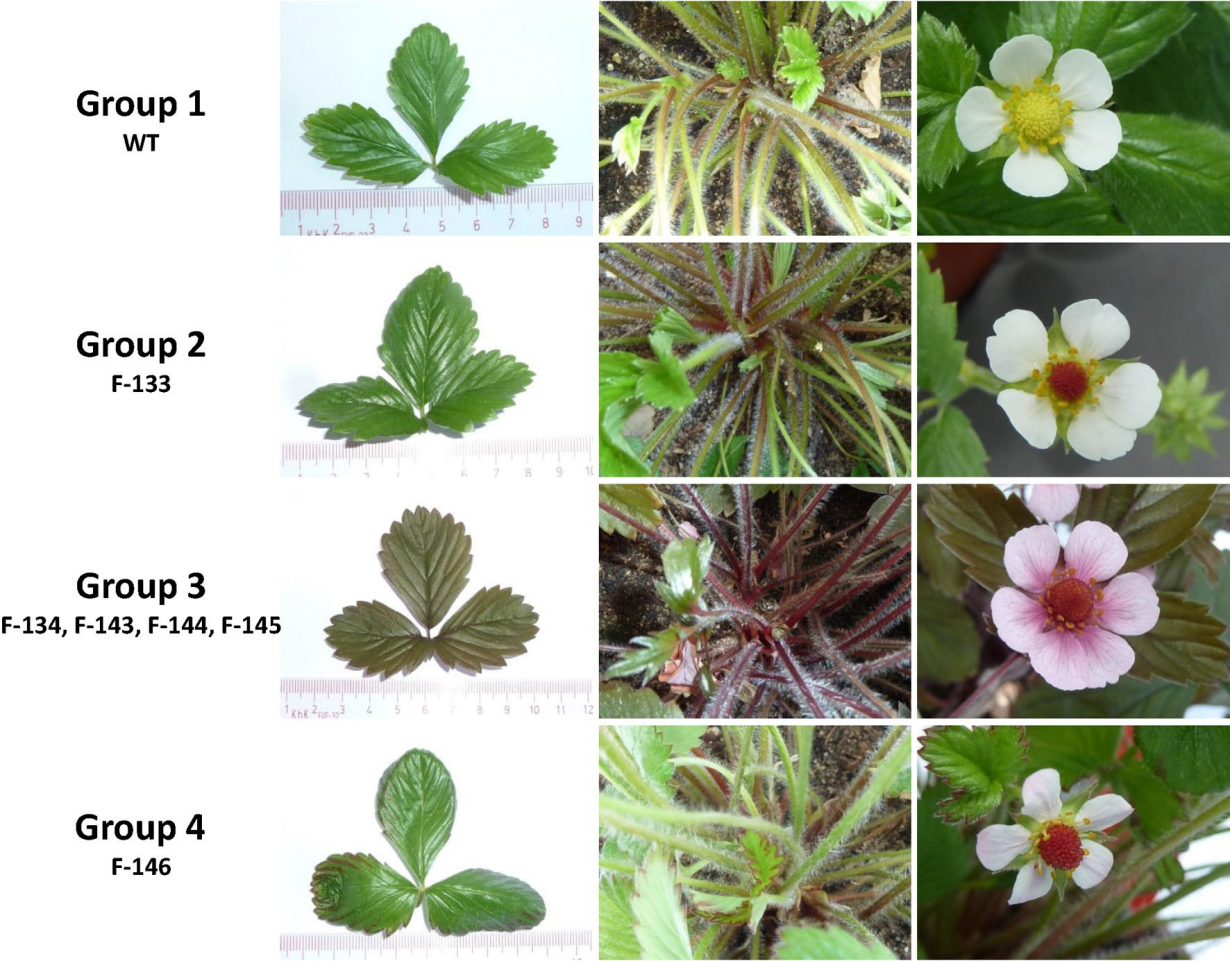
Phenotypic comparison of the transgenic strawberry plants transformed with p9N::35S-MdMYB10-GFP. Greenhouse grown plants of the transgenic strawberry plants (groups 2, 3 and 4) and the non-transgenic wild type (WT) cv. ‘Rügen’ were evaluated on their coloration of their leaves (*left*), petiole (*middle*), and flower organs (*right*). All phenotypes could be allocated to four different groups (Table 3)

Five clones for each of the transgenic lines and the wt were rooted, transferred to the greenhouse and grown under normal light and temperature conditions. These plants were evaluated on visible signs (red tissue coloration) of *MdMYB10* expression. Based on their coloration of different plant organs, all plants transformed with p9N::35S-MdMYB10-GFP43 were classified into four different groups, with the wt cv. ‘Rügen’ as group 1 (Table 3). A complete red coloration of all plant organs as expected in the case of constitutive overexpression of *MdMYB10* was only found in the group 3 clones F-134, F-143, F-144, and F-145 (Fig. 3). Lines of groups 2 and 4 were characterized by green leaves (with red edges in group 4), yellow stamens, and white (group 2) or very slightly red (group 4) colored petals (Table 3). Whether these differences in tissue coloration are due to differences in the *MdMYB10* mRNA transcript levels or not is beyond the scope of this study.

**Table 3:** Phenotypic classification of transgenic strawberry lines transformed with p9N::35S-MdMYB10-GFP43

Group	Genotype	Upper leaf surface	Petiole	Sepal	Petal	Stamen	Carpel	mg Cy_equ_/g FW
1	Rügen	Green	Light red, green	Light green	White	Yellow	Light yellow	Below detection level in leaves and petals
2	F-133	Dark green	Light red, green	Light green	White	Yellow	Pink	Below detection level in leaves and petals
3	F-134	Light red, to red	Red to dark red	Light red to red	Light red to red	Light red to red	Pink	3 ± 0.1 in leaves and petals
	F-143							
	F-144							
	F-145							
4	F-146	Green with reddish edges	Light red with reddish spots	Light red	Light red	Yellow	Light pink	Below detection level in leaves and petals

**Fig. 4:**
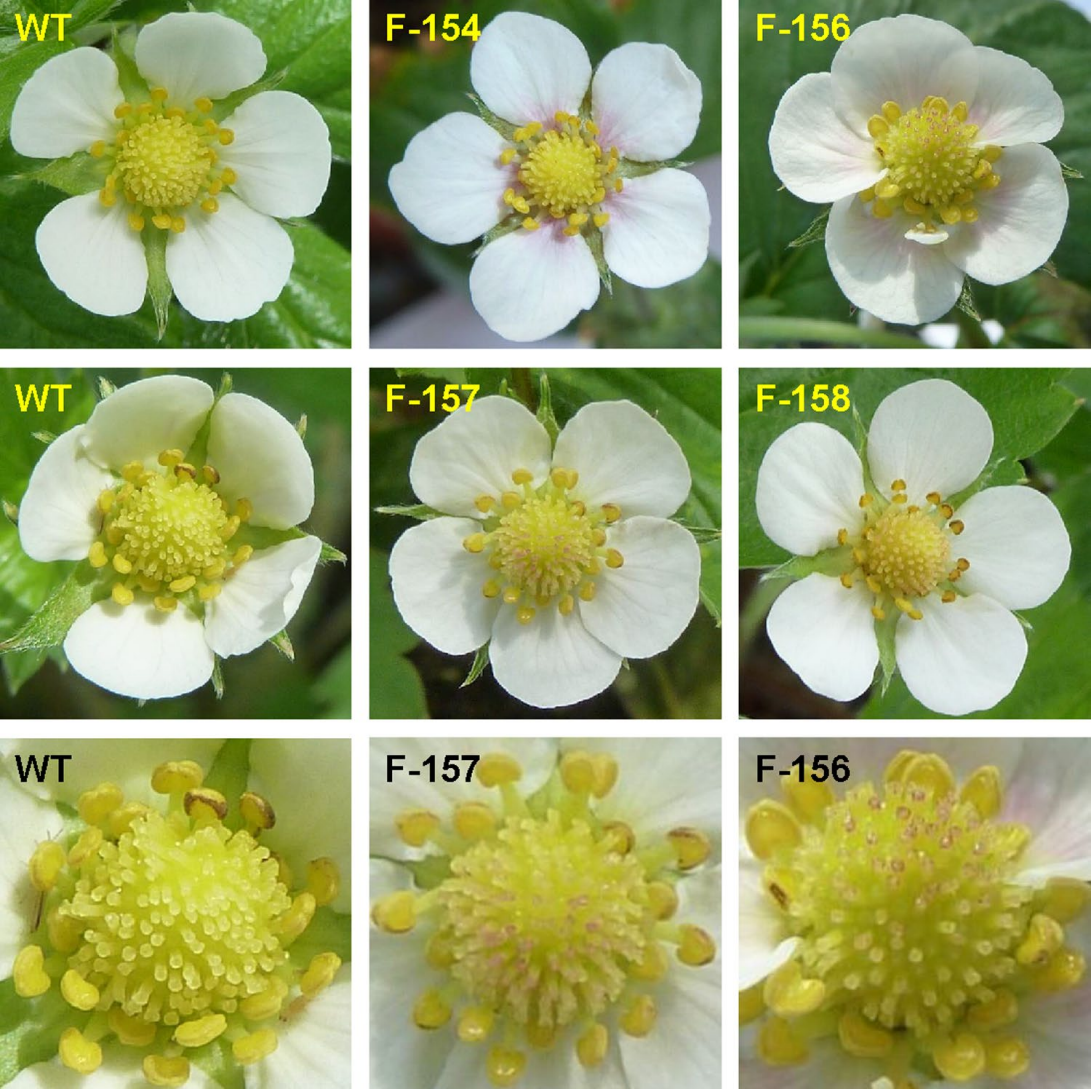
Phenotypic comparison of the flower organs of the transgenic strawberry plants transformed with p9N::F3*′*H-MdMYB10-GFP43. Coloration of flower organs was evaluated on greenhouse grown plants of the non-transgenic wild type (WT) cv. ‘Rügen’ (*left*) and transgenic strawberry plants (*middle* and *right*). Red coloration was sometimes found at the base of transgenic petals (*top middle* and *right*) or on transgenic stigmas (*middle* and *bottom, middle* and *right*)

**Fig. 5:**
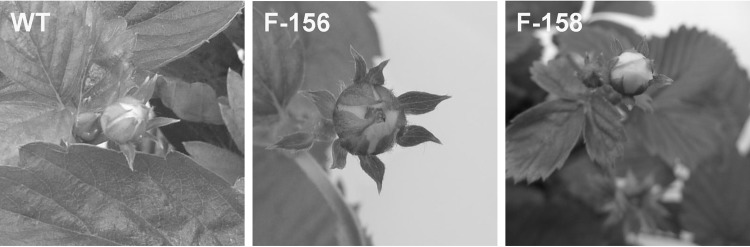
Phenotypic comparison of the closed flower buds of transgenic strawberry plants transformed with p9N::F3′H-MdMYB10-GFP43. Coloration of sepals was evaluated on greenhouse grown plants of the non-transgenic wild type (WT) cv. ‘Rügen’ (*left*) and transgenic strawberry plants (*middle* and *right*). Red coloration was sometimes found in transgenic sepals (*middle* and *right*)

Much less red coloration of plant organs was found for lines transformed with p9N::F3*′*H-MdMYB10-GFP43. These plants could hardly be distinguished from wt plants. A slight red coloration was sometimes found at the base of petals or on transgenic stigmas (Fig. 4). Sometimes red coloration was also found on sepals of very young and still closed flower buds of transgenic lines (Fig. 5). Such red coloration of sepals, petals and stigmas was never found on wt plants.

Leaf, stem, flower, and flower bud samples of all transgenic lines and the wt were investigated on GFP fluorescence using UV light microscopy. GFP specific fluorescence signals were neither detectable in tissue strongly expressing *MdMYB10* nor in green tissue in any of the lines.

### Evaluation of tissue specific expression of the *MdMYB10*/*GFP43* fusion gene

**Fig. 6:**
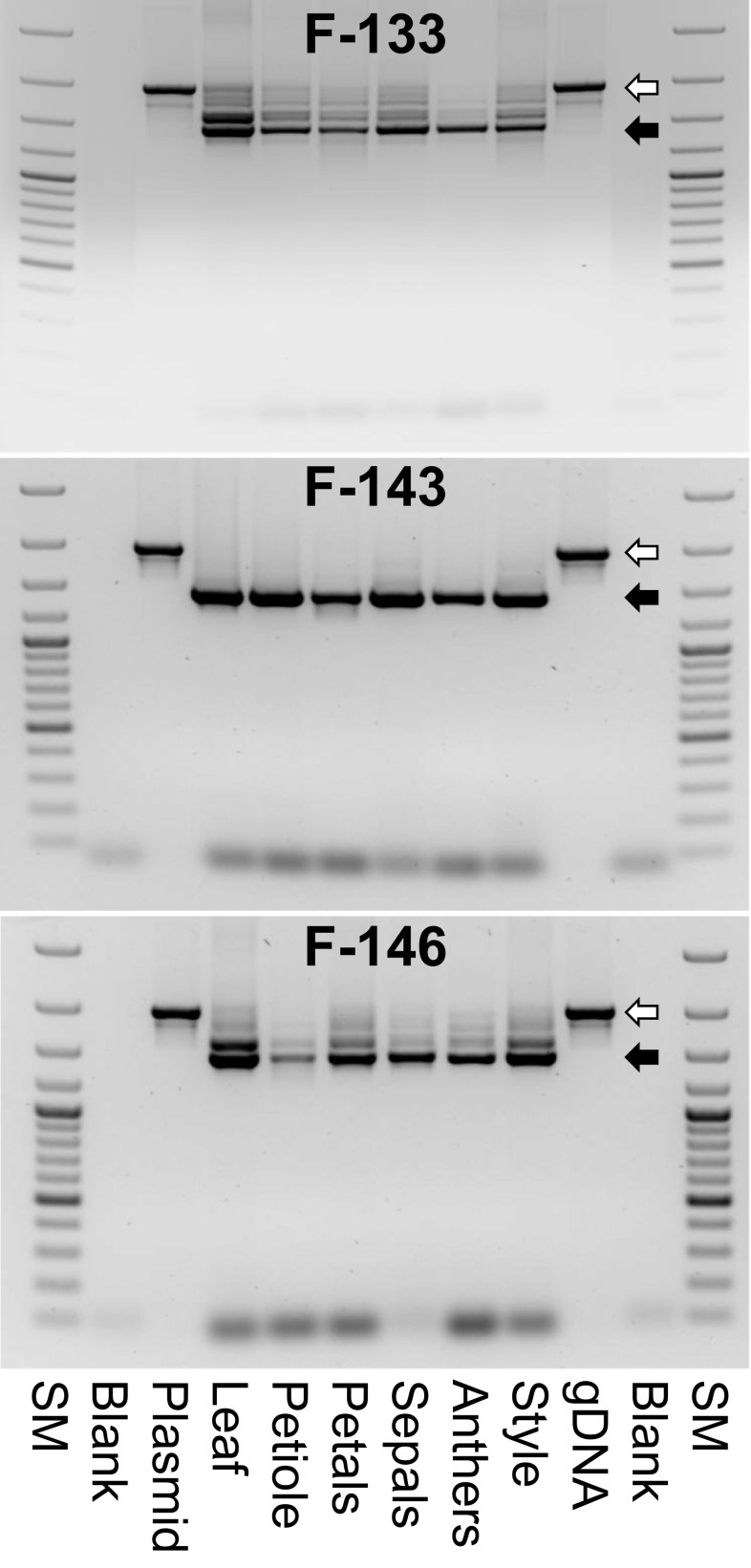
Evaluation of *MdMYB10*/*GFP43* transgene expression in different tissues of p9N::35S-MdMYB10-GFP transgenic strawberry plants. SM, Gene Ruler™ 100 bp DNA Ladder Plus (ThermoFisher Scientific, Schwerte, Germany) with fragment sizes of 100, 200, 300, 400, 500 (*bold*), 600, 700, 800, 900, 1000 (*bold*), 1200, 1500, 2000, and 3000 bp; Blank, H_2_O instead of DNA used as negative control; Plasmid, DNA of the plasmid used for plant transformation as positive control. The white arrow marks the 1974 bp PCR fragment expected for genomic DNA containing both introns. The *black arrow* marks the 1462 bp PCR fragment expected for mRNA without introns. Amplicons between these two fragments indicate the presence of incorrectly spliced versions of the *MdMYB10*/*GFP43* gene (1651 bp with Intron 2, but without intron 1 and 1785 bp with Intron 1, but without Intron 2, respectively)

Different tissues of the transgenic strawberry lines were tested on *MdMYB10*/*GFB43* transgene expression using the primers MYB_ATG and GFP_RR. Lines F-133, F-143 and F-146 were selected as representative examples for the phenotypic classes 2, 3 and 4 (Table 3) of the transgenic lines expressing *MdMYB10-GFP43* gene construct driven by the CaMV 35S promoter. All tissues of all plants expressed a fragment of the expected size of ~1.5 kbp (Fig. 6). However, transgenic plants of the phenotypic classes 2 and 4 expressed additional fragments of larger sizes. Based on their fragment lengths these amplicons seem to represent different versions of the *MdMYB10*/*GFP43* fusion gene which were incompletely spliced. Incorrect splicing of the two introns of the *MdMYB10*/*GFP43* fusion gene could be reason for the phenotypic differences observed between the transgenic lines of different phenotypic classes.

**Fig. 7:**
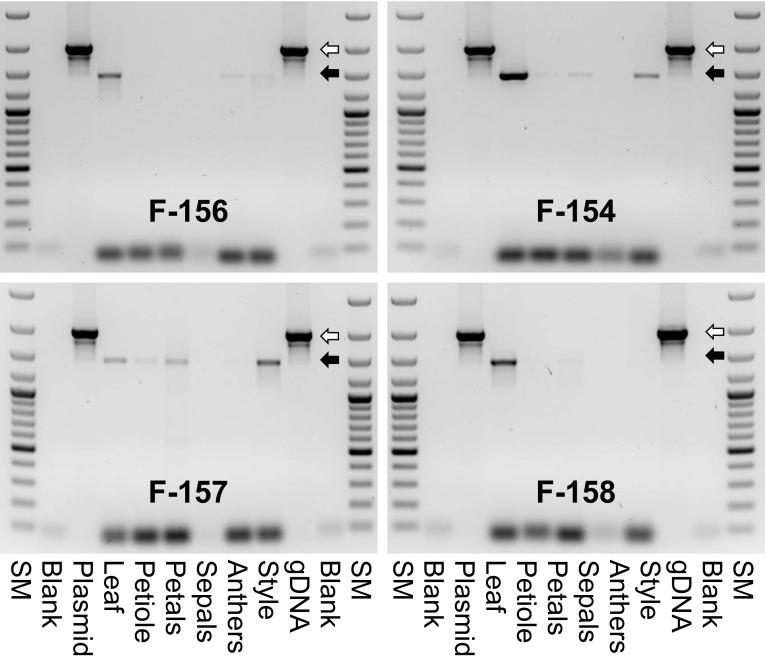
Selected examples of the evaluation of MdMYB10/GFP43 transgene expression in different tissues of p9N::F3*′*H-MdMYB10-GFP43 transgenic strawberry plants. SM, Gene Ruler™ 100 bp DNA Ladder Plus (ThermoFisher Scientific, Schwerte, Germany) with fragment sizes of 100, 200, 300, 400, 500 (*bold*), 600, 700, 800, 900, 1000 (*bold*), 1200, 1500, 2000, and 3000 bp; Blank, H_2_O instead of DNA used as negative control; Plasmid, DNA of the plasmid used for plant transformation as positive control. The *white arrow* marks the 1974 bp PCR fragment expected for genomic DNA containing both introns. The *black arrow* marks the 1462 bp PCR fragment expected for mRNA without introns

Subsequently the expression of the *MdMYB10-GFP43* gene in different tissues of plants containing the gene driven by the F3′H promoter was also tested. The expression pattern of the transgene varied between the different transgenic lines, but also within the plants of the same line. A few selected examples of this investigation are shown in Fig. 7. Expression was always detectable in leaf tissue. In all other tissues the transgene was occasionally expressed. In summary, it can be stated that the expression of the *MdMYB10-GFP43* gene under control of the F3′H promoter was always detectable in cases where red colored tissue was investigated, but sometimes the transcript could also been detected in non-red tissues. Incorrect splicing was not as frequently found in transgenic lines transformed with the F3*′*H-MdMYB10-GFP43 gene construct.

## Discussion

This study served the purpose of testing an *MdMYB10*/*GFP43* fusion gene for suitability as a non-destructive visual reporter gene for functional genomics studies in transgenic strawberry plants. GFP protein is frequently used as marker in functional plant science (Gunadi et al. 2016; Halfhill et al. 2003; Harper et al. 1999; Richards et al. 2003; Stewart 2001; Zhang et al. 2002) but requires special equipment for its detection. Over-expression of the *MdMYB10* gene, in contrast, usually leads to strong red tissue coloration in different species of the Rosaceae plant family (Dixon et al. 2013; Espley et al. 2007, 2012; Medina-Puche et al. 2014) and should therefore allow the visual and non-destructive detection of promoter activity in different plant tissues throughout the entire life cycle of the plant. However, artifacts resulting in anthocyanin formation due to stress response or other environmental cues cannot be excluded. Therefore *GFP43* gene was fused to *MdMYB10* to allow the double-checking of red coloured tissue microscopically on *GFP* expression. Our construct provokes the expression of a protein which contains the MYB-type transcription factor MdMYB10 of apple at its N-terminus and a codon optimized version of GFP at its C-terminus.

The *MdMYB10*/*GFP43* fusion gene was tested in combination with two plant promoters for proof-of-concept. The CaMV 35S promoter was used because of its constitutive nature and the usually high expression level of genes driven by it (Odell et al. 1985). In this case, a high expression of *MdMYB10*/*GFP43* was expected to result in a strong red coloration of transgenic tissues as described for strawberry plants expressing the *MdMYB10* gene under the control of a natural mutant version of its own promoter and its own terminator (Kortstee et al. 2011). The other putative promoter was obtained from the ornamental plant *C. sulphureus* by cloning the 5′-flanking regions of the *F3′H* gene of the flavonoid pathway. An ornamental plant, rather than the native *F3′H* promoter of *Fragaria*, was chosen to avoid undesired silencing events caused by sequence similarities.

In *C. sulphureus*, two closely related genes of the flavonoid pathway have been identified, which encode enzymes introducing a second hydroxyl group in the B-ring of flavonoids and chalcones, respectively. However they showed interesting tissue specific differences in their expression pattern, because *CH3H* was primarily expressed in flowers and seedlings (Schlangen et al. 2010). *F3′H*, in contrast seemed to be constitutively and moderately expressed (Schlangen et al. 2010; Yuan et al. 2014).

From the transgenic plants obtained, only those carrying the *MdMYB10*/*GFP43* fusion gene under the control of the CaMV 35S promoter revealed strongly red colored tissues as expected. However, not all the plants showed uniform coloration. Whereas four out of six transgenic lines had complete dark red coloration in all organs (leaves, stems, petals, sepals, pistils, stamens), the other two showed deep red color only in the pistils, but no or only faintly colored leaves, petals, stems and/or stamen. Incorrect splicing of the *MdMYB10*/*GFP43* fusion gene is proposed as one possible reason for the differences found in tissue coloration (see Fig. 6). However, anthocyanin production was observed in all our transgenic lines albeit in varying intensities which is in contrast to Kortstee et al. (2011) where not all plants carrying the *MdMYB10* gene showed coloration.

The transgenic lines carrying the *MdMYB10*/*GFP43* fusion gene under the control of the *F3′H* promoter showed much less coloration. Although leaves, stems and receptacles remained uncolored as in the wt plants, all transgenic lines obtained showed a faint coloration in the tissues of the stamen, petals and/or sepals. A varying occurrence of *MdMYB10*/*GFP43* gene transcripts was observed in the different plant tissues of these lines. This confirms that the isolated 1.7 kb long 5′-flanking region is sufficient to control the expression of the reporter gene as expected from sequence analysis and comparison with other *F3′H* promoters (Sun et al. 2015; Yuan et al. 2014). However, the control by the *F3′H* promoter was not as effective as desired as our isolated putative *F3′H* promoter was not active in all the lines in the organs. This could indicate that the *F3′H* promoter is a weak promoter in comparison with the CaMV 35S promoter. It can be excluded that the observed effects were based on a negative interference by the native *Fragaria F3′H* promoter that is also present in the transgenic plants because of the low sequence identity (approx. 40%) to the *Fragaria F3′H* promoter and very short stretches of sequence identity found (below 8 nucleotides).

Another explanation could result from the fact that the *F3′H* promoter, as flavonoid formation in general, could be influenced by UV (Harborne and Williams 2000; Sun et al. 2015). As the plants were grown under greenhouse conditions it is possible that we did not completely exploit the potential of the *F3′H* promoter. Analysis of the 5*′*-flanking region of *F3′H* predicted several cis-regulatory elements which suggest the regulation of expression mainly in response to light and biotic/abiotic stress factors (e.g. high/low temperature, elicitors, drought, wounding, abscisic acid and ethylene) which is in accordance with the findings for e.g. the promoter regions of *Vitis F3′H* (Sun et al. 2015) or *Tulipa F3′H* (Yuan et al. 2014) and *F3′H* expression data of *Sorghum bicolor* (Shih et al. 2006).

In contrast to the *MdMYB10* reporter gene, no GFP signal was microscopically detected although *GFP43* was detected at transcriptional level by RT-PCR in the transgenic plants. GFP fusion proteins have however been previously reported to show divergent success when transformed in plants. Fusion with a gene of interest as N- or C-terminal tag in some cases have no effect and in some others affect expression of *GFP* itself or the fused gene. The expression of *anthocyanin2* (*AN2*) fused with *GFP* under control of the CaMV 35S promoter showed an altered phenotype but GFP could not be detected by confocal microscopy or immunoblot analysis in 35S:AN2-GFP lines (Quattrocchio et al. 2013). This was interpreted as a result of too low transgene expression or undesired fusion protein cleavage yielding unstable GFP fragments. Whether an N-terminal fusion of *GFP43* to *MdMYB10* would lead to a microscopically detectable GFP expression or not, cannot be stated. At this stage, it remains open why the *GFP43* component of the fusion gene turned out to be inactive. Splicing variations cannot be blamed for the non-functionality as GFP activity could also not be detected in F143 and the *F3′H-promoter* lines, in which GFP was correctly spliced. Although this part of the study remained therefore unachieved, the reporter gene construct allowed us to evaluate the suitability of the *MdMYB10*/*GFP43* gene as reporter gene for promoter analysis.

## Conclusion

Our studies have demonstrated the limited suitability of the *MdMYB10* gene as a reporter gene for promoter studies because in the case of a weak promoter, differences between the transgenic plants and the wild type lines can hardly be distinguished, particularly in tissues showing a strongly colored background such as green leaves. In addition it has to be stated that the benefit of a visualization without the need for fluorescence or light imaging can probably not completely compensate for possible undesired side effects such as influence on anthocyanin formation caused by biotic and abiotic factors (Harborne and Williams 2000) or possible physiological effects of anthocyanins on the plants (Taylor and Grotewold 2005).

## Electronic supplementary material

Below is the link to the electronic supplementary material.

Supplementary material 1 (DOCX 14 KB)

## Abbreviations

CDS: Coding sequence
CH3H: Chalcone 3-hydroxylase
CaMV: Cauliflower mosaic virus
Cy_equ_: Cyanidin equivalent
F3*′*H: Flavonoid 3*′*-hydroxylase
GFP: Green fluorescent protein
Md: *Malus domestica*
