# Supplementary material for: Evaluation of a MdMYB10/GFP43 fusion gene for its suitability to act as reporter gene in promoter studies in Fragaria vesca L. ‘Rügen’
Source: Plant Cell Tissue Organ Cult. 2017 May 16;130(2):345–56. doi: 10.1007/s11240-017-1229-0 (PMC5515962; doi:10.1007/s11240-017-1229-0)
Supplement: Supplementary file 1 — Supplementary material 1 (DOCX 14 KB) [file 11240_2017_1229_MOESM1_ESM.docx]

**Supplementary material**

**Suppl. Table S1:** Primers used for promoter isolation

| Name | Sequence (5′-3′) | Application |
| --- | --- | --- |
| CH3H.GSP1.a | AAGTACGTACAGTAGTAAGGCAGTTAG | Genome Walking |
| CH3H.GSP2.a | GGGTAGAGTAGTAGGGGTAGAATAGTCAT |  |
| CH3H.GSP1.b | ATTGCCGAAATTAATCTCAACTCAGTACTG |  |
| CH3H.GSP2.b | TGCTCTTTGTTTAAGCACTTGGTAAAG |  |
| F3′H.GSP1.a | CTGGCGAAATTCGCGTCATGAGTTTTC |  |
| F3′H.GSP2.a | GAGTGCTTTGCCGGAAAATAGGTG |  |
| F3′H.GSP1.b | GGGCCAGTTGGAAAAGTGTGCTATAAT |  |
| F3′H.GSP2.b | GAAAGGTTGGTAGAACGTGTGGAAGAA |  |
| F3′H.GSP1.c | CCAGATGTCATCAAAAAGGTCCAAAAC |  |
| F3′H.GSP2.c | CCAATGTCCGATGCTTGTAACTTCAAC |  |
| Pro.CH3H.F | ATGGTATGAGATTATGGCTCAGAT | Amplification of 5′ flanking region |
| Pro.CH3H.R | AGTAGTAGGGGTAGAATAGTCAT |  |
| Pro.F3′H.F | TTAGGGTATAATTCCCGATTCGTTC |  |
| Pro.F3′H.R | AGAATCGTCATTTCGGATACGG |  |

**Isolation of the 5′-flanking region of *chalcone 3-hydroxylase* (*CH3H*) of *Cosmos sulphureus***

The 5′-flanking region *CH3H* was isolated as described for *F3′H* in the Materials and Methods section. First primer pairs were designed on basis of the sequence of *CH3H* (GenBank: FJ216429) (Schlangen et al. 2010) and further primers from the obtained sequences (Suppl. Table S1). Finally, a DNA clone containing 1433 bp (*CH3H*) (Gen bank: KU508432)of the 5′-flanking region was obtained with the primers Pro.CH3H and Pro.F3′H, respectively.
